# Vitamin D receptor is present on the neuronal plasma membrane and is co-localized with amyloid precursor protein, ADAM10 or Nicastrin

**DOI:** 10.1371/journal.pone.0188605

**Published:** 2017-11-27

**Authors:** Erdinç Dursun, Duygu Gezen-Ak

**Affiliations:** Brain and Neurodegenerative Disorders Research Laboratory, Department of Medical Biology, Cerrahpasa Faculty of Medicine, Istanbul University, Istanbul, Turkey; Torrey Pines Institute for Molecular Studies, UNITED STATES

## Abstract

Our recent study indicated that vitamin D and its receptors are important parts of the amyloid processing pathway in neurons. Yet the role of vitamin D receptor (VDR) in amyloid pathogenesis is complex and all regulations over the production of amyloid beta cannot be explained solely with the transcriptional regulatory properties of VDR. Given that we hypothesized that VDR might exist on the neuronal plasma membrane in close proximity with amyloid precursor protein (APP) and secretase complexes. The present study primarily focused on the localization of VDR in neurons and its interaction with amyloid pathology-related proteins. The localization of VDR on neuronal membranes and its co-localization with target proteins were investigated with cell surface staining followed by immunofluorescence labelling. The FpClass was used for protein-protein interaction prediction. Our results demonstrated the localization of VDR on the neuronal plasma membrane and the co-localization of VDR and APP or ADAM10 or Nicastrin and limited co-localization of VDR and PS1. E-cadherin interaction with APP or the γ-secretase complex may involve NOTCH1, NUMB, or FHL2, according to FpClass. This suggested complex might also include VDR, which greatly contributes to Ca^+2^ hemostasis with its ligand vitamin D. Consequently, we suggested that VDR might be a member of this complex also with its own non-genomic action and that it can regulate the APP processing pathway in this way in neurons.

## Introduction

Nobel Prize awardee in 1928, Adolf Windousin had this honor for the discovery of vitamin D hormone. This unique secosteroid hormone has significant roles in many cellular mechanisms and diseases, yet used to be neglected or ignored by the researchers particularly when its role in brain is the issue. Eyles et.al have meticulously reviewed the evidence that vitamin D differentiates brain cells, regulates axonal growth and calcium signaling directly in the brain, modulates the production of brain-derived reactive oxygen species and stimulates the production of neurotrophic factors which are relevant to the variety of neuropsychiatric conditions and also neurodegenerative disorders [1]. Our *in vivo* and *in vitro* studies [2–8] and other publications in the field support the effect of vitamin D on neurodegeneration and Alzheimer’s disease (AD), but the cellular mechanisms involved in this effect is still unknown. The intriguingly complex effects of vitamin D receptor (VDR) silencing or 1,25-Dihydroxyvitamin D_3_ treatments on α-, β-, γ-secretases, APP and the obvious result on amyloid production that we demonstrated in our previous study [9] indicated that some of the vitamin D/its receptors depended regulations might not be explained solely by transcriptional regulation. This suggestion led us to investigate the localization of VDR in the neuronal plasma membrane, possibly as a part of a signal relaying mechanism or as a partner of the enzyme complexes (i.e: APP processing enzymes). A few studies suggested that the classical VDR, which has a role in gene transcription, can also be near to or associated with caveolae that is present in the plasma membrane of osteoblasts [10, 11]. Bartoccini et al. demonstrated that the presence of VDR in the lipid rich microdomains within the nuclear membrane. This particular microdomains resembles those found in the plasma membrane, in developing hippocampal neurons [12]. To our knowledge no studies have reported the localization of VDR on the neuronal plasma membrane.

On the other hand, amyloid precursor protein (APP) processing is a process that is not fully understood, but is also suggested as a process that has crucial roles in development and maintenance of cell life. Particularly in neurons, this process involves many aspects of a cell life including synaptic transmission, synaptic plasticity and immune response [13]. APP processing is the main event in production of amyloid beta peptide in AD pathogenesis. APP processing involves three enzyme complexes: α- β- or γ-secretase. α-secretase is composed of a disintegrin and a metalloprotease (ADAM) like either ADAM10, 9 or 17. β-secretase is composed of an aspartic protease like beta amyloid cleaving enzyme (BACE1, 2). γ-secretase complex is composed of different proteins including Presenilin-1 (PS1), Presenilin-2 (PS2), anterior pharynx-defective 1 (Aph-1), presenilin enhancer 2 (Pen2) and nicastrin [14, 15]. Though, we should note that APP is not the only substrate that is processed by these complexes and these enzyme complexes are also involved in the neurodevelopmental pathways and the psychiatric disorders [16]. So far, a few studies demonstrated the co-localization of VDR with caveolin 1 [17], and others showed APP to be localized with caveolin 1 [18] and gave us the opportunity to speculate that VDR and APP are found in lipid rafts in close proximity. Considering that vitamin D is a secosteroid that has a broken cholesterol chain and is a ligand of VDR [19], the binding of APP to cholesterol [20] may be relevant. Given the chemical similarity between cholesterol and vitamin D, it is possible that vitamin D may directly interact with APP. So far, pro-vitamin D has been suggested to be a membrane component in its evolutionary process due to its chemical structure, which lacks two hydrogens compared to cholesterol [21].

In light of these suggestions, the present study primarily focused on the localization of VDR in neurons and its interaction with amyloid pathology-related proteins. The other suggested vitamin D receptor, a multifunctional protein known as endoplasmic reticulum protein 57/60 kDa (ERp57 or ERp60), a chaperone, or protein disulfide isomerase A3 (PDIA3) and, recently, vitamin D membrane associated, rapid-response, steroid-binding protein (1,25-MARRS) [22], was suggested to be localized on the plasma membrane of intestinal cells [23, 24] and was also investigated in our study.

## Results

### Localization of VDR on the neuronal plasma membrane

**Fig 1:**
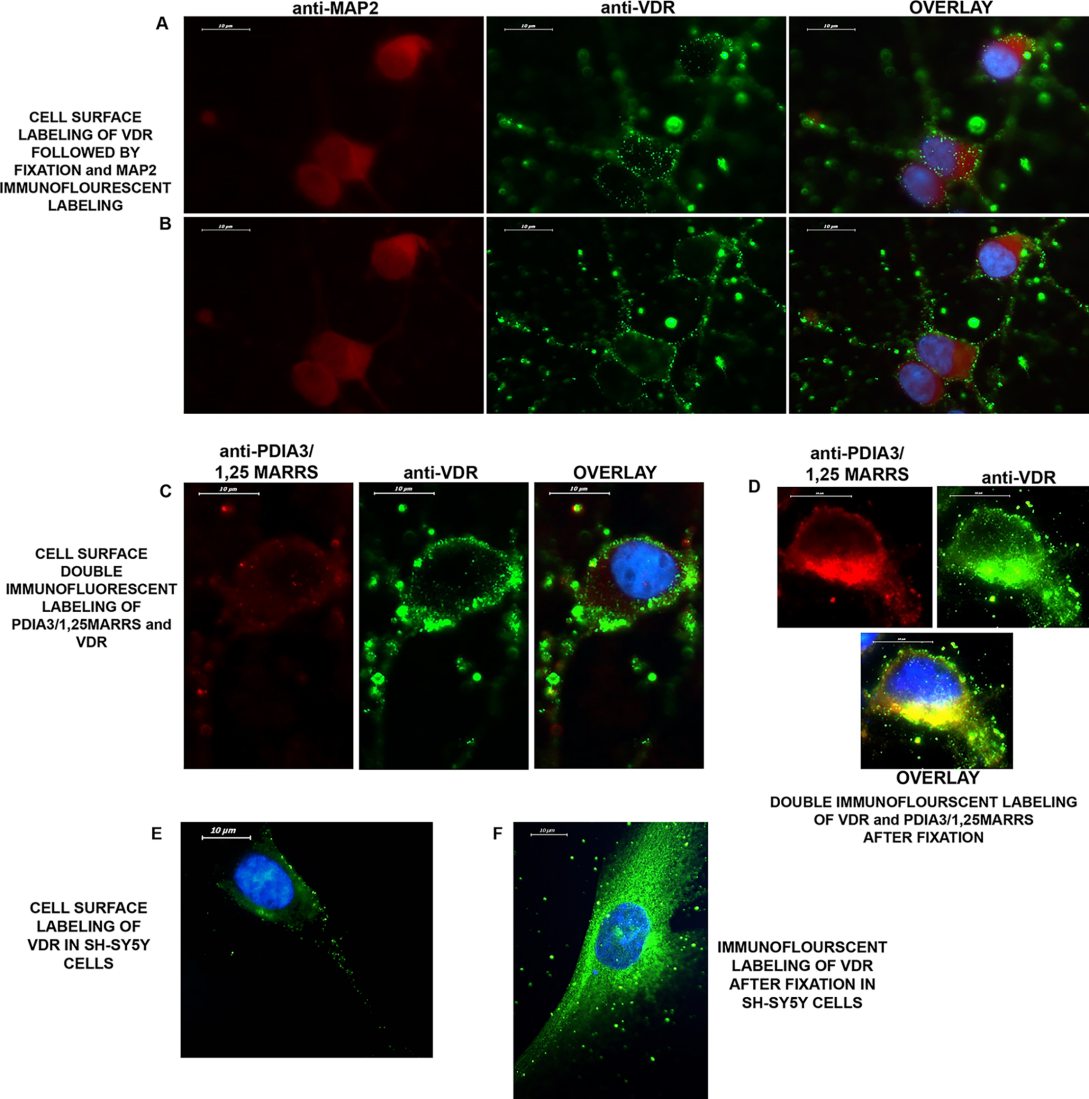
The expression of VDR and PDIA3/1,25MARRS in cortical neurons. **A-B)** Cell surface staining of VDR (green) in live neurons, followed by fixation and immunofluorescence labeling of MAP2 (red) as a neuronal marker, 100x. The micrographs were taken from the same areas with different levels of focus to demonstrate the different localization of VDR on the neuronal plasma membrane. *A)* VDR protein is localized on the plasma membrane of the soma; *B)* VDR protein is localized on the plasma membranes of neurites. The 3D image of staining was obtained via confocal microscopy (63x), and the video is presented in S1 Video. **C)** VDR (green) and PDIA3/1,25MARRS (red) cell surface staining with live neurons via double immunofluorescence labelling, 100x. The localization of PDIA3/1,25MARRS on the neuronal plasma membrane was very limited. **D)** Double immunofluorescence labelling of VDR and PDIA3/1,25MARRS in fixed and permeabilized neurons, 100x. PDIA3/1,25MARRS was localized in the cytoplasm and in the endoplasmic reticulum (ER). Given its strong reactivity in a certain area in the cytoplasm and its known role as an ER chaperone, the localization was considered to be in the ER. VDR is located in the nucleus, cytoplasm, ER and axon hillock. VDR and PDIA3/1,25MARRS might co-localize in the cytoplasm, especially in the ER. **E)** VDR (green) cell surface staining with live SH-SY5Y cells via immunofluorescence labelling, 100x. **F)** Immunofluorescence labelling of VDR in fixed and permeabilized SH-SY5Y cells, 100x.

Cell surface staining with a VDR antibody in live neurons showed, to our knowledge for the first time, that VDR is present on the neuronal cell surface (Fig 1, S1 Video). On the other hand, cell surface staining of PDIA3/1,25MARRS did not indicate a presence of the protein as much as VDR on the plasma membrane (Fig 1C). Cell surface staining with a VDR antibody in live SH-SY5Y cells were confirmed the presence of VDR on the plasma membrane (Fig 1E and 1F).

### Possible co-localization of VDR with target proteins

**Fig 2:**
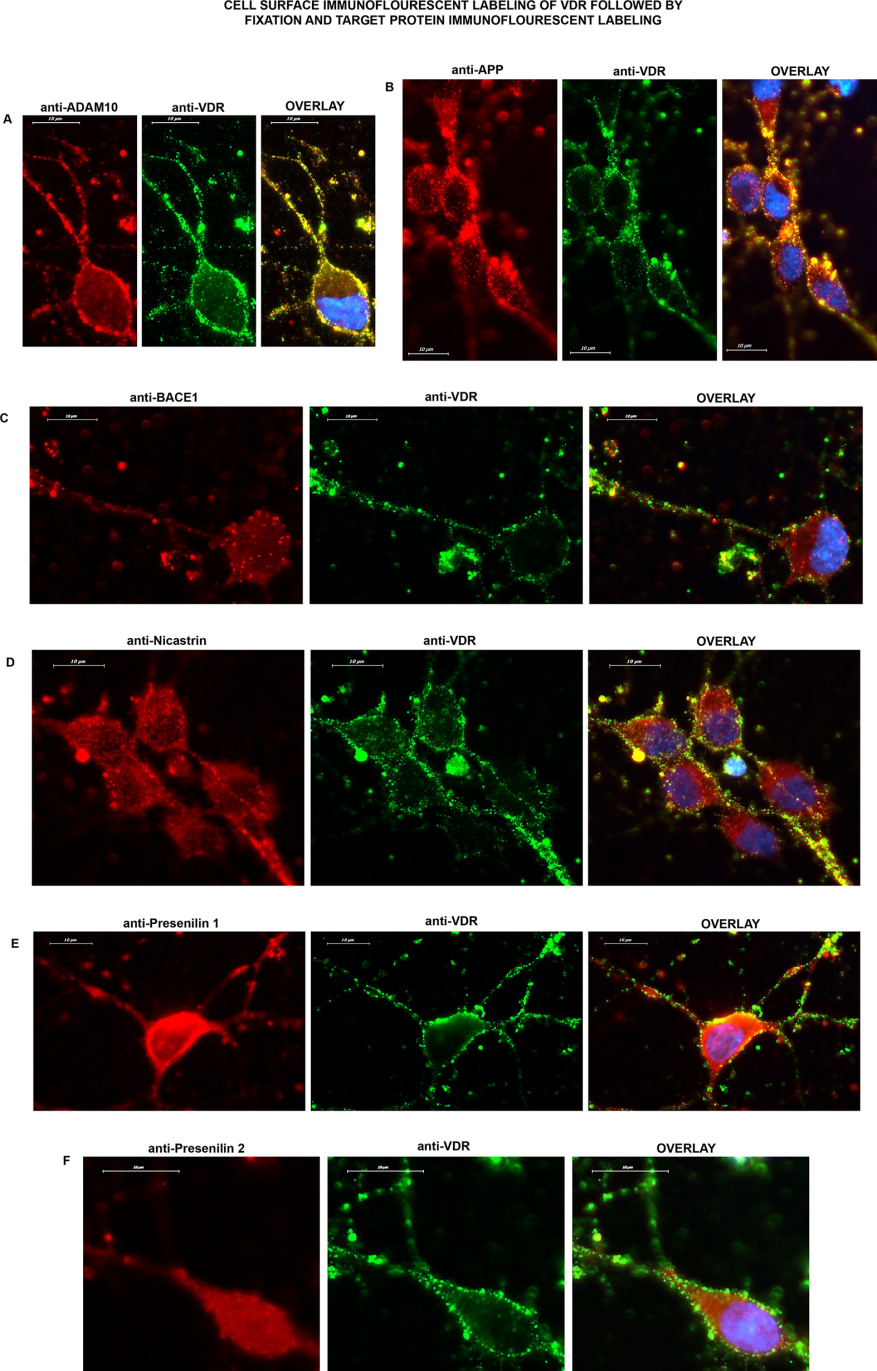
Cell surface staining of VDR (green) on live neurons followed by fixation and immunofluorescence labeling of target proteins (100x): **A)** ADAM10; **B)** APP; **C)** BACE1; **D)** Nicastrin; **E)** Presenilin 1; **F)** Presenilin 2. Overlay images indicate the possible co-localization of VDR/ADAM10 (A) or VDR/APP (B) or VDR/Nicastrin (D) or limited co-localization of VDR/ Presenilin 1 (E) on the neuronal plasma membrane. Overlay images do not indicate co-localization for VDR/BACE1 (C) or VDR/Presenilin 2 (F) on the neuronal plasma membrane.

The possible co-localization of VDR with APP processing proteins was another question to be explored. Cell surface staining of VDR on live neurons followed by fixation and immunofluorescence labeling of target proteins showed the possible co-localization of VDR/ADAM10, VDR/APP, VDR/Nicastrin or the limited co-localization of VDR/PS1 on the neuronal plasma membrane (Fig 2).

### PPI prediction results

**Fig 3:**
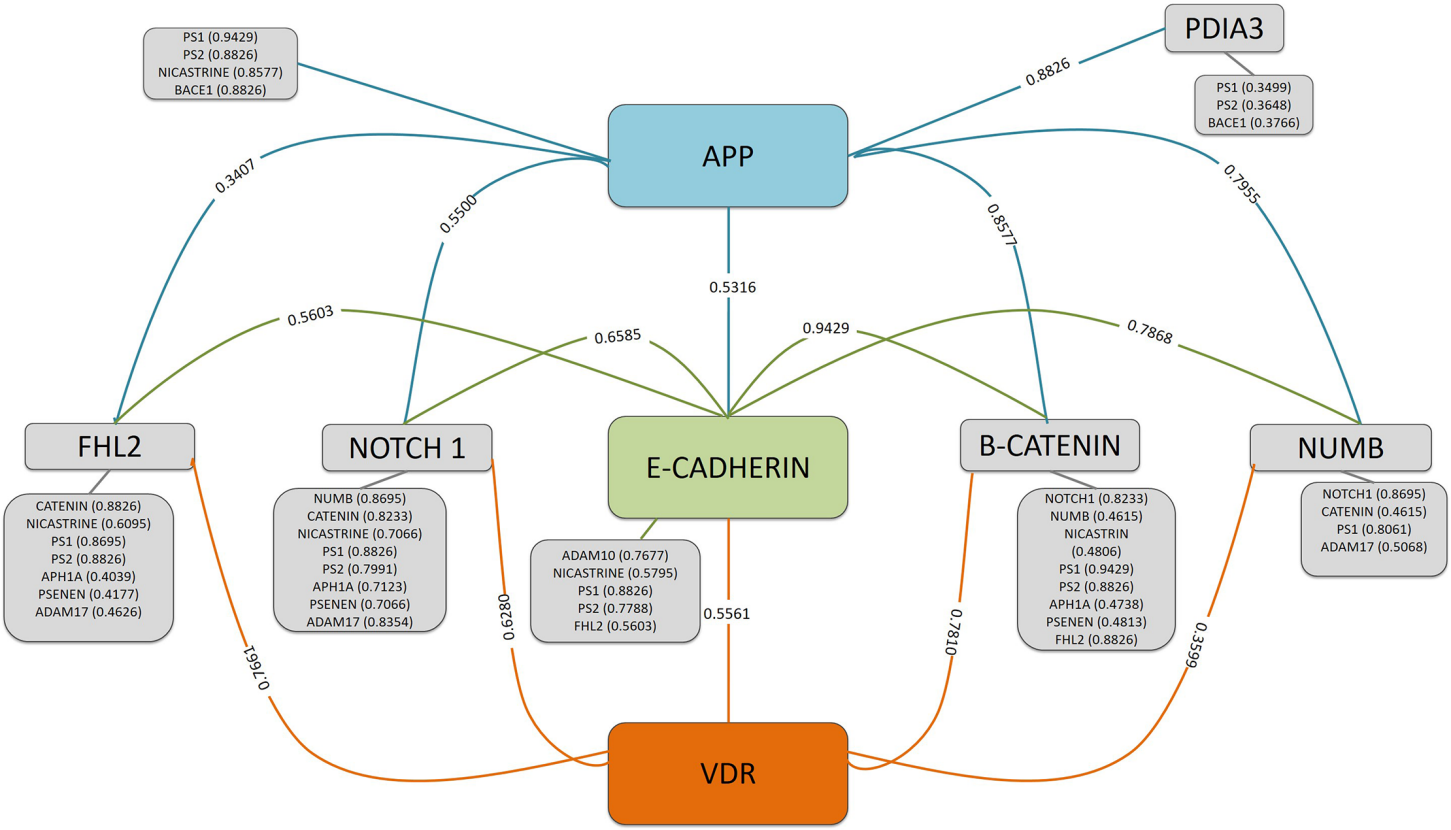
Summary of FpClass PPI prediction tool data. The FpClass PPI prediction tool was used to identify partner proteins for both APP and VDR. The tool predicted 1133 partners for APP and 583 partners for VDR. An analysis of the FpClass tool data indicated that 153 of these partners interacted with both APP and VDR. A total of 153 proteins were classified according to their functions in S1 Table. Five of these proteins (NUMB, catenin (CTNNB1), NOTCH1, E-cadherin (CDH1), and FHL2) were membrane or membrane-related proteins. These 5 proteins were used for further analyses with the target proteins (PS1, PS2, Nicastrin, BACE1, ADAM10) and PDIA3. The software predicted 5244 partners for these proteins, and the proteins that are the most relevant to plasma membrane interactions are presented in the figure with their PPI total score.

Given that cell surface staining indicated the localization of VDR on the neuronal plasma membrane, and its possible co-localization with the substrate APP, APP and VDR proteins were analyzed for PPI prediction and the software predicted 1133 partners for APP and 583 partners for VDR. The mutual proteins for both APP and VDR were chosen, and the list of proteins is presented in S1 Table. Five of these proteins (NUMB, catenin (CTNNB1), NOTCH1, E-cadherin (CDH1), and FHL2) were membrane or membrane-related proteins. These 5 proteins were used for further analysis with the target proteins (PS1, PS2, Nicastrin, BACE1, and ADAM10) and PDIA3. The software predicted 5244 partners for these proteins, and most related proteins are provided in Fig 3 with the PPI prediction total score.

## Discussion

The present study primarily focused on the possible non-genomic action of vitamin D. Our initial thought was that a non-genomic action might require the localization of VDR on the neuronal plasma membrane. The other suggested vitamin D receptor, PDIA3/1,25MARRS, was suggested to be localized on the plasma membrane of intestinal cells [23, 24]. Therefore, we labelled VDR and PDIA3/1,25MARRS on the surface of living neuronal cells and determined that VDR was abundantly localized on the neuronal membrane. Note that we have confirmed the specificity of the anti-VDR antibody which was used in immunofluorescent labeling and cell surface staining, in our previous study [9]. The presence of VDR on the plasma membrane was also confirmed in SH-SY5Y cells. The function of VDR on the neuronal surface was then evaluated. Immuno-labelling results showed the co-localization of VDR especially with APP and ADAM 10 and Nicastrin. We then examined the possible interaction between VDR and these proteins by using data from a PPI prediction tool.

The results of our previous study [9] led to questions about the VDR-dependent regulation of APP processing proteins. One potential regulatory mechanism was transcriptional regulation by VDR [9], and the other was the possible rapid response (RR) activity of the VDR. Some studies indicate that the RR properties, which are defined as non-genomic effects of steroid hormones, are mediated by receptors located near or associated with the plasma membrane or its caveolae components [25–27]. For example, the suggested RR role of vitamin D involves the rapid intestinal absorption of Ca^+2^, the secretion of insulin by pancreatic cells or the opening of voltage-gated Ca^+2^ and Cl^-^ channels in osteoblasts [26]. A few studies suggested with solid evidence that the classical VDR, which has a role in gene transcription, can also be near to or associated with caveolae present in the plasma membrane and can mediate the RR role of vitamin D [10, 11]. Our fluorescence and confocal microscopy results are the first to demonstrate the localization of VDR on the plasma membranes of live neurons. The antibody that we used for cell surface labelling recognizes the ligand binding domain of the VDR. Therefore, the ligand binding site of VDR may be localized on the outer site of the plasma membrane. This is also relevant if VDR, which is also responsible for Ca^+2^ homeostasis in different cell types, mediates RR after binding to its ligand on the neuronal plasma membrane.

On the other hand, the cell surface staining of PDIA3/1,25MARRS did not support the studies in intestinal cells that suggested that PDIA3/1,25MARRS was a plasma membrane receptor for vitamin D [23, 24]. The low immunoreactivity of PDIA3/1,25MARRS compared with the VDR immunoreactivity might indicate that neurons tend to use VDR for a possible non-genomic action of vitamin D.

After we detected the presence of VDR on the plasma membrane, the question arose as to whether VDR directly interacted with the proteins involved in APP processing including PS1, PS2, NICASTRIN, ADAM10, BACE1 and the APP substrate on neuronal plasma membranes. We labelled VDR again with a cell surface staining protocol in live neurons, and then double immunofluorescence labelling was performed one by one for target proteins. Interestingly, we observed the possible co-localization of VDR with APP and ADAM10 and Nicastrin on the neuronal plasma membrane.

The next question that emerged from our results was whether VDR interacts directly or indirectly with APP or ADAM10 or Nicastrin. A PPI prediction tool, FpClass, was used to uncover possible interactions or partner proteins. The data that we gathered from FpClass indicated no direct interaction between VDR and APP or VDR and ADAM10 or VDR and Nicastrin. However, the results indicated that VDR and APP interacted directly with 153 mutual proteins with a variety of functions (S1 Table). Because our main focus was the plasma membrane, we selected the proteins that interact with both VDR and APP and function as membrane or membrane-related proteins. The prominent proteins were identified as E-cadherin (encoded by CDH1), β-catenin (encoded by CTNNB1), NOTCH1, NUMB and Four and a half LIM domains 2 (FHL2 or DRAL) (Fig 3). The following discussion explains how this indirect interaction between VDR and the APP processing pathways might involve these five proteins.

Like N-cadherin, E-cadherin is also a Type I classical cadherin and is expressed in the CNS. Types I and II cadherins are localized in synaptic compartments of neurons that border the neurotransmitter release zones. In addition, N- and E-cadherins formed heterodimers with an intermediate affinity compared to their respective homodimers when both were present in proximity. They are associated with catenins and synaptic complex components, particularly at excitatory synapses [28, 29]. The catenins are cytosolic proteins that link cadherins to actin and microtubules to provide mechanical support [30]. N-cadherin expression facilitates the homodimerization of APP, and the dimerization of APP modulates Aβ production [31]. The blockade of N-cadherin function was thought to accelerate Aβ synaptotoxicity, and patients with Alzheimer’s disease have increased levels of proteolytically-cleaved N-cadherin C-terminal fragment 1 [32]. PS1 cleaves a variety of type I membrane proteins, including APP, NOTCH1, and N- and E-cadherin [33, 34]. Furthermore, Wnt/ β-catenin signaling was also reported to be negatively regulated by PS1 [35]. ADAM10 was suggested to cleave E-cadherin, which is mainly expressed in satellite glial cells [36]. Our data from the FpClass PPI prediction tool indicate that ADAM10 might interact with E-cadherin and ADAM17 rather than the other secretases or APP. This result might also indicate that the molecule that joins ADAM10 to the APP processing complex is E-cadherin (Fig 3).

AD pathogenesis was suggested to include alterations in the proteolysis of NOTCH1 by γ-secretase [37]. The relationship between NOTCH and E-cadherin has been demonstrated in a couple of examples. NOTCH forms a complex with E-cadherin, a transmembrane component of the adherens junction. The depletion of E-cadherin downregulated NOTCH signaling. This finding indicated that NOTCH signaling might require the cadherin-mediated adherens junction in *Drosophila*. Additionally, NOTCH1 was suggested to physically interact with ZO-1, a constituent of the adherens junction [38]. NOTCH enhanced cell adhesion via the induction of E-cadherin in small cell lung cancer [39]. The monomeric PTB-containing adaptor NUMB regulates NOTCH/Sanpodo traffic and prevents NOTCH and Sanpodo localization to the cell surface, thus suppressing NOTCH signaling [40]. One study showed that mouse NUMB and NUMB-like are required for the maintenance of radial glial adherens junctions. NUMB accumulates in the apical end-feet and interacts with cadherins [41].

FHL2 has been detected in the cell membrane, cytoplasm and nuclear compartments of the cell. It is hypothesized that FHL2 acts as a protein interaction platform. The binding of proteins to FHL2 was suggested to make proteins more accessible for post-translational modification or to affect their stability. FHL2 is thought to be present in multi-protein complexes where it can either promote or prevent the interaction between other proteins [42]. FHL2 was reported to interact directly with PS2, and the activation of PS2 is facilitated by FHL2 [43]. FHL2 was suggested to interact with ADAM-17 and regulate ADAM-17 localization. FHL2 can interact with a member of nuclear receptor superfamily androgen receptor (AR). VDR is also a member of this family [42]. However, no studies have examined the interaction between FHL2 and VDR.

In summary, we can support the theory that interactions exist among APP, γ-secretase complex, E-cadherin, NOTCH1, NUMB, β-catenin and FHL2 with FpClass results and previous findings. E-cadherin appears to be an essential component of this complex because all of these proteins have a possible interaction with E-cadherin rather than N-cadherin, which only interacted with catenin, PS1, and FHL2, according to FpClass data. Possible interactions between E-cadherin and VDR were indicated by FpClass data, and the possible interaction was also supported by the following studies. Vitamin D increased the expression of E-cadherin and decreased the level of vimentin, which was associated with the elevated expression of VDR. Moreover, vitamin D reduced the expression of transcription factors of the epithelial-mesenchymal transition, such as slug, snail, and β-catenin, in ovarian cancer cells [44]. It was suggested that vitamin D regulates TH expression directly and that this regulation involves N-cadherin as a plausible mediator [45]. Vitamin D and VDR affect Wnt signaling through a direct interaction with β-catenin, resulting in the attenuation of growth in colon cancer cells. Vitamin D was reported to promote the differentiation of these cells via the induction of E-cadherin and the inhibition of β-catenin signaling. The study demonstrated that vitamin D induced the expression of adhesion proteins and promoted the translocation of nuclear β-catenin and ZO-1 to the plasma membrane [46]. β-catenin regulated cell proliferation and adherens junction formation, which are essential for re-epithelialization following wounding, and requires vitamin D, VDR and a calcium sensing receptor [47]. Vitamin D-depleted rats showed induced skeletal muscle atrophy and a downregulation of cleaved NOTCH1 expression [48].

In addition to all of these regulations and interactions, the most important factor might be the Ca^+2^. Increased intracellular Ca^+2^ levels enhance production of Aβ1–42 probably via Ca^+2^ influx that modulates cleavage of APP by β- and γ-secretases [49]. A study reported that PS1 adopts a pathogenic closed conformation within “minutes of Ca^+2^ influx”, suggesting a Ca^+2^ dependent mechanism [50]. PS1 was suggested to regulate Ca^+2^ homeostasis and Ca^+2^ release from intracellular stores [35]. Cell adhesion properties of the cadherins depend on Ca^+2^. The ability of the members of the cadherin superfamily to mediate adhesion mainly depends on the EC domains (Extracellular Cadherin repeats) and requires Ca^+2^. Ca^+2^ removal leads to the disorientation of the ectodomains and results in a loss of adhesive structure [29]. Although function of E-cadherin depends on extracellular Ca^+2^ levels, it also depends on both Ca^+2^ influx through membrane channels and the release of Ca^+2^ from internal Ca^+2^ stores [51]. All of these clues given above might be in accordance with RR properties of VDR-vitamin D.

In summary, our results demonstrated the localization of VDR on the neuronal plasma membrane and the co-localization of VDR and APP or ADAM10 or Nicastrin and limited co-localization of VDR and PS1. This co-localization might be in accordance with the strong evidence for the RR properties of VDR on the plasma membranes reported in other cell types. It also supports the effect of VDR on APP processing that we previously reported [9]. VDR might be in close proximity or be a part of complex that involves APP processing enzymes. Further analysis like co-immunprecipitation or FRET might be needed to show whether the interaction between co-localized proteins is direct or indirect. The calcium-dependent E-cadherin interaction with APP or the γ-secretase complex may also involve NOTCH1, NUMB, or FHL2, according to FpClass data. This complex might also include VDR, which greatly contributes to Ca^+2^ hemostasis with its ligand vitamin D. Given that, we speculated that VDR might be a member of this complex also with its own non-genomic action and that it can also regulate the APP processing pathway in this way in neurons.

## Materials and methods

### Preparation of primary cortical neuron cultures

Primary cortical neuron cultures were prepared from the neocortex of embryonic day 16 Sprague-Dawley rat embryos as previously described [52–54]. The neuron/glia ratio of the cultures was evaluated by IF labeling with neuronal (Millipore MAB2300) and glial (Invitrogen AB5804) markers using The Leica Application Suite Image Overlay Software as previously described [52, 54]. The glia ratio to total cell number of the cultures was 25%. The study was approved by the Animal Welfare and Ethics Committee of Istanbul University with the numbers 24.02.2011/13, 26.07.2012/102, and the procedures that involved experimentation on animal subjects were done in accord with both the guide of Istanbul University and with the National Research Council's guide for the care and use of laboratory animals.

### SH-SY5Y cultures

To confirm the presence of VDR on the plasma membrane besides primary cortical neurons we additionally used SH-SY5Y cells. Briefly, the cells were plated in DMEM media containing 2mM L-glutamine, 10% FBS and were incubated at 37°C and 5% CO_2_ in a humidified atmosphere. These cells were used for the cell surface staining and immunoflouresent labelling of VDR.

### The specificity of anti-VDR antibody

The specificity of the anti-VDR antibody (ab3508, Abcam) which was used in immunofluorescent labeling and cell surface staining method, was checked with silencing of VDR expression via siRNA treatment in primary cortical neurons in our previous study [9]. The immune labeling of VDR protein in VDR silenced neurons was decreased almost 70% in ourprevious study [9]. This silencing efficiency was also consistent with mRNA and western blot results in our several studies [3, 9, 55]. Given the reliability of this anti-VDR antibody, it was chosen for cell surface staining method.

### Immunofluorescent labeling of VDR and PDIA3/1,25MARRS

Double immunofluorescent labeling was used for determining the cellular localization of VDR or PDIA3/1,25MARRS protein. 3.7% paraformaldehyde was used for fixation, and then neurons were blocked with 30% goat serum in 0.02% T-PBS for 1 h at room temperature. After that, they were incubated with primary antibodies overnight at 4°C. They were further processed with corresponding secondary antibody labeled with Alexa Fluor 488 (A11034, ThermoFisher) in the dark for 1 h at room temperature. After labelling with the first primary antibody, another blocking step with 10% goat serum was performed. Blocking was then followed by incubation with the second primary antibody for 2 h at room temperature. Neurons were then labeled with corresponding secondary antibody tagged with Alexa Fluor 568 (ab175703, Abcam) for 1 h at room temperature. Primary antibodies for double immunofluorescent labeling were as follows: rabbit polyclonal antibody to VDR -ChIP Grade- (ab3508, Abcam) followed by mouse monoclonal antibody to PDIA3/1,25MARRS (Erp57/Grp58) (NBP2-36765, Novus). The Leica Application Suite Image Overlay Software (Leica Microsystems Ltd, Heerbrugg, GE) was used to obtain overlay images. Negative controls for immunofluorescent staining were also included by omitting primary antibodies.

### VDR cell surface staining in live neurons and double immunofluorescent labeling with target proteins

To demonstrate the possible localization of VDR in the plasma membrane, we performed cell surface staining as previously described [56] with an anti-VDR antibody on the cortical neuron cultures that were plated onto poly-L-ornithine covered 13-mm cover glasses No:1 (631–0149, VWR) that were cultured as 4 cover glasses per one well of 6-well plates as described in the Neuronal Cultures section. Briefly, prior to fixation, each cover glass with primary cortical neurons was placed into a well in a 24-well plate (Corning CLS3524. Corning Inc. New York, USA). The cultured neurons were incubated with rabbit polyclonal antibody to VDR -ChIP Grade- (at 1:50; ab3508, Abcam) that was diluted 1:50 in neurobasal medium NBM (GibcoBRL 21103–049) that contained 0.02 M HEPES (Sigma H0887) and 1% BSA (GibcoBRL 15260–037, New York, USA) for 1 h at room temperature. The neuronal cultures were fixed with 3.7% paraformaldehyde in phosphate buffer, pH 7.4, and were incubated with goat anti-rabbit secondary antibody labeled with Alexa Fluor 488 (at 1:100; A11034, ThermoFisher) for 45 minutes. Negative controls for immunofluorescent staining were also established by omitting the primary antibody. DAPI was used as a counterstain. Images were taken with a Leica DMIL inverted fluorescence microscope with a CCD camera. The Leica Application Suite Image Overlay Software (Leica Microsystems Ltd., Heerbrugg, GE) was used to obtain overlay images of Alexa Fluor 488-conjugated anti-rabbit IgG and DAPI. Assessments were also repeated with the Leica TCS SPE confocal system.

VDR cell surface staining was performed with each target protein to determine possible co-localization. Briefly, VDR cell surface staining and another blocking step with 10% goat serum were performed. The neurons were then treated with a second primary antibody for 2 h at room temperature to detect a target protein involved in secretase systems. Neurons were then labeled with a corresponding secondary antibody tagged with Alexa Fluor 568 for 1 h at room temperature. Primary antibodies for double immunofluorescent labeling were as follows: rabbit polyclonal antibodies to ADAM10 (at 1:100, ab19026, Abcam), APP (at 1:50, NB300-308, Novus), Presenilin 1 (at 1:50, NBP1-76792, Novus), Nicastrin (at 1:50, NBP1-77269), rabbit monoclonal antibody to Presenilin 2 (at 1:50, NB110-57435, Novus), and mouse monoclonal antibody to BACE1 (at 1:50, MA1-177, ThermoFisher).

### Protein-protein interaction (PPI) prediction with FpClass

To evaluate the potential interaction of VDR and target proteins that are related to amyloid pathology, a web-based prediction tool was used (https://omictools.com/protein-protein-interaction-prediction-category). The FpClass tool (https://omictools.com/fpclass-tool) is a data mining-based method for proteome-wide protein-protein interaction (PPI) prediction. Protein IDs of target proteins were used for the analysis of PPI prediction [57].

## Supporting information

S1 Table The FpClass PPI prediction tool was used to identify partner proteins for both APP and VDR. The tool predicted 1133 partners for APP and 583 partners for VDR. An analysis of the FpClass tool data indicated that 153 of these partners interacted with both APP and VDR. A total of 153 proteins were classified according to their functions.
(DOCX)

S1 Video 3D confocal image. The cell surface staining of VDR (green) in live neurons followed by fixation and immunofluorescence labeling of MAP2 (red) as a neuronal marker, 63x.
(MP4)
