# Supplementary material for: Vitamin D receptor is present on the neuronal plasma membrane and is co-localized with amyloid precursor protein, ADAM10 or Nicastrin
Source: PLoS One. 2017 Nov 27;12(11):e0188605. doi: 10.1371/journal.pone.0188605 (PMC5703467; doi:10.1371/journal.pone.0188605)
Supplement: S1 Table — The tool predicted 1133 partners for APP and 583 partners for VDR. An analysis of the FpClass tool data indicated that 153 of these partners interacted with both APP and VDR. A total of 153 proteins were classified according to their functions. (DOCX) [file pone.0188605.s001.docx]

**S1 Table:** The FpClass PPI prediction tool was used to identify partner proteins for both APP and VDR. The tool predicted 1133 partners for APP and 583 partners for VDR. An analysis of the FpClass tool data indicated that 153 of these partners interacted with both APP and VDR. A total of 153 proteins were classified according to their functions.

| Protein Translation/ Modification | MEMBRANE/membrane related proteins | TRANSCRIPTION FACTORS/  REGULATION | | NFĸbeta pathway | Nuclear receptors | Cell cycle/ Apoptosis | Cytokines/ Immune response | Intracellular signalling pathways | | Chaperons | Proteosome pathway | Cytoskeleton |
| --- | --- | --- | --- | --- | --- | --- | --- | --- | --- | --- | --- | --- |
| RPL11  EIF2AK2  SUMO1 | NUMB  CTNNB1  NOTCH1  CDH1  GHR (Somatotropin receptor)  FHL2 | JUN  FOS  NR3C1  ATF2  ATF3  ETS1  ETS2  ATF4  JUNB  POLR2A  MED28  NCOR2  SIRT1  RUNX1  RUNX2  EP300  NCL  RELB  PAX6  FANCA  SP1  NFYA  ELK1  TBP  HDAC4  HDAC6  HDAC7  NFE2L2  BCL6 | IRF1  IRF3  HIF1A  PARP1  EDF1  HNF1A  TOP1  FOXO1  NFE2  NKX2-1  NFATC2  CREBBP  SMYD2  SNAI1  YBX1  XRCC6  GTF2B  SMARCA4  SMARCE1  RAD51  SPEN  CTBP2 (Synaptic ribbon)  RFWD2  KHDRBS1  BAZ2A | RELA  TAB2  IKBKB  IKBKG  NFKB1  CHUK  BCL3 | PPARG  AHR  ESR1 (ERalpha)  NR2F2  NCOA1  NCOA3 | CDK1  CDK5  PTEN  TP53  RB1  TP53BP2  CDKN1A  CDC25C  MYB  MYC  NDN  RB1CC1  WWOX  BRCA1  BCL2  BCL2L1  DAXX  CCND1  CCNE1  NOL3  ING1  PLK1 | TGFBR1  TRAF6  IL8  PTMA  IFNB1 | SRC  MAPK1  MAPK8  MAPK9  MAPK11  MAPK14  STAT1  STAT3  PIAS1  GSK3B  PIN1  ABL1  PRKCA  PRKCD  CSNK1A1  CSNK1D  CSNK2A1  CSNK2A2  CSK  SMAD2  SMAD3 | ZFYVE9  STRAP  SRGAP3  PTK2  PIK3R1  VRK1  AXIN1  RPS6KA1 RPS6KA3  S100B  TNIK  STK4  STK11  PPP1CC (PP-1G)  TXN  CTSL1 | HSP90AA1  HSP90B1  HSF1  HSPA1B  HSPB1 | COPS5  UBE2I  PSME3  PSMC5  MDM2 | VIM  ACTB |
